# The influence of effective microorganisms (EM) and yeast on the degradation of strobilurins and carboxamides in leafy vegetables monitored by LC-MS/MS and health risk assessment

**DOI:** 10.1007/s10661-015-5022-4

**Published:** 2015-12-30

**Authors:** Elżbieta Wołejko, Bożena Łozowicka, Piotr Kaczyński, Magdalena Jankowska, Jolanta Piekut

**Affiliations:** Faculty of Civil and Environmental Engineering, Bialystok University of Technology, Wiejska Str. 45 E, 15-351 Bialystok, Poland; Plant Protection Institute – National Research Institute, Regional Experimental Station, Pesticide Residue Laboratory, Chełmońskiego Str. 22, 15-195 Bialystok, Poland

**Keywords:** Fungicides, Microorganisms, Lettuce, LC-MS/MS, Chronic risk analysis

## Abstract

The aim of this study was to determine the behaviour of strobilurin and carbocyamides commonly used in chemical protection of lettuce depending on carefully selected effective microorganisms (EM) and yeast (Y). Additionally, the assessment of the chronic health risk during a 2-week experiment was performed. The statistical method for correlation of physico-chemical parameters and time of degradation for pesticides was applied. In this study, the concentration of azoxystrobin, boscalid, pyraclostrobin and iprodione using liquid chromatography–mass spectrometry (LC–MS/MS) in the matrix of lettuce plants was performed, and there was no case of concentration above maximum residues levels. Before harvest, four fungicides and their mixture with EM (1 % and 10 %) and/or yeast 5 % were applied. In our work, the mixtures of 1%EM + Y and 10%EM + Y both stimulated and inhibited the degradation of the tested active substances. Adding 10%EM to the test substances strongly inhibited the degradation of iprodione, and its concentration decreased by 30 %, and in the case of other test substances, the degradation was approximately 60 %. Moreover, the addition of yeast stimulated the distribution of pyraclostrobin and boscalid in lettuce leaves. The risk assessment for the pesticides ranged from 0.4 to 64.8 % on day 1, but after 14 days, it ranged from 0.0 to 20.9 % for children and adults, respectively. It indicated no risk of adverse effects following exposure to individual pesticides and their mixtures with EM and yeast.

## Introduction

Against plant diseases such as downy mildew, grey mould and powdery mildew (Gilardi et al. 2008; Kretschmer et al. 2009). it is common to use fungicidal agents that assist in the elimination of pathogens and which have severe adverse effects on crop yield and quality (Camoutsis et al. 2010; Łozowicka et al. 2012; Łozowicka et al. 2014).

The innovative generation of fungicides, which includes such groups as strobilurins and carboxamides, have a broad spectrum of fungicide which are intensively employed throughout the world to fight against highly destructive plant pathogens, such as *Botrytis cinerea, Sclerotinia spp., Leveillula taurica,* or *Spherotheca macularis* (Mendoza et al. 2005; Kretschmer et al. 2009; Łozowicka 2015) which are found on many kinds of fruits and vegetables (Łozowicka 2015). Boscalid and iprodione belong to the carboxamide group of pesticides, and they show a biological mode of action consisting in the inhibition of the enzyme succinate-ubiquinone reductase, also known as complex II, in the mitochondrial electron transport chain (Mendoza et al. 2005; Camoutsis et al. 2010). Moreover, pyraclostrobin and azoxystrobin belong to strobilurins, a new class of fungicides included in the quinone outside inhibitors (QoI) group, which have a novel mode of action, and are very safe from an environmental point of view. The strobilurin fungicides are synthetic active ingredients similar to the natural strobilurin A, produced by the strobilurus tenacellus fungus. The effectiveness of strobilurins lies in their inhibition of the mitochondrial respiration of the fungus (Manna et al. 2013).

The intensive degradation of active substances of fungicide will depend heavily on their physico-chemical properties and the plants on which application has been made as well as environmental factors (temperature, light or moisture in the air). In spite of the difficulties in precisely determining the impact of all factors influencing the degradation of the plant protection product (PPP), the chemical structure of the active substance is one of the most important factors determining the rate of degradation, and on this basis, one can specify certain regularities (Kah et al. 2007; Swarcewicz and Gregorczyk 2012). The high value of octanol-water partition coefficient (log *P)* corresponds with the low aqueous solubility and high value of bioconcentration factor (BCF) (e.g. for pyraclostrobin BCF = 706, for azoxystrobin BCF = low risk) (EPI 2011). Azoxystrobin, boscalid, pyraclostrobin and iprodione are contact pesticides; thus, they stay on the surface of leaves for a longer time.

According to many authors, the microorganisms may also significantly contribute to the degradation of the active substance (Kah et al. 2007; Aktar et al. 2009; Manna et al. 2013). Biodegradation of fungicide using effective microorganisms (EM) seems to be interesting and reasonable because as shown by Zhou et al. (2009). they are widely used in the cultivation of crops, vegetables and animal husbandry. EM contains selected species of microorganisms including predominant populations of lactic acid bacteria, yeasts, low density of photosynthetic bacteria, actinomyces and other types of natural microorganisms. These microorganisms are mutually compatible with one another and they can coexist in liquid culture (Higa 1994). According to Deiana et al. (2002). microorganisms are useful in eliminating problems associated with the use of chemical fertilizers and pesticides and therefore are now widely applied in natural farming and organic agriculture. On the other hand, El-Tarabily and Sivasithamparam (2006) also indicate a positive effect of yeast both on acceleration of the development of plants and on their protection against fungal pathogens. According to Meinhardt and Klassen (2009). *Saccharomyces cerevisiae* are particularly active in the quick conversion of sugars into alcohol and carbon dioxide, thus contributing to the limited availability of nutrients for other organisms inhabiting the plant organs. In addition, they are capable of producing so-called “toxin killers” which, as protein complexes, exhibit very strong inhibitory properties of pathogens located in the same environment.

Vegetables play an important role in the human diet because they supply components regulating biological processes, thereby contributing to maintaining health in an important way. Lettuce is one of many vegetables, which is very often used in the diet, served to children and adults as a salad or as an addition to sandwiches. According to WHO (2003). the mean daily consumption of lettuce in Europe is 22.5 g, which represents about 6.5 % of the total dietary intake of vegetables. The global trend indicates an increase in pesticide use in vegetables crops, and hence the prevalence of residue in plants. It is a result of the emergence of pathogens races resistant to pesticides, which in turn can cause a serious threat to food security, and thus to consumers. The maximum residue level (MRL) in food for children is 0.01 mg kg^−1^, while for the rest of the population is set at 10 mg kg^−1^ MRL e.g. for iprodione.

The aim of this study was to determine the behaviour of strobilurin and carbocyamides commonly used in chemical protection of lettuce depending on carefully selected effective microorganisms (EM) and yeast (Y). Additionally, the assessment of the chronic health risk during a 2-week experiment was performed. The statistical method for correlation of physico-chemical parameters and time of degradation for pesticides was applied. Moreover, the time in order to determine the interval between the application of PPP and harvest of the lettuce leaves, required for food for children, e.g. at the pesticide residues concentration levels below 0.01 mg kg^−1^ was calculated.

## Materials and methods

### Samples and reagents

In this study, 240 samples of lettuce were investigated. Pesticide-free lettuce was used as blank to spike for the validation process. All reagents used were analytically graded acetonitrile, acetone, hexane and methanol pesticide residue provided by J.T. Baker (Deventer, Holland). Magnesium sulphate anhydrous, sodium chloride, trisodium citrate dihydrate (Na_3_C_6_H_5_O_7_ · 2H_2_O) and disodium hydrogen citrate sesquihydrate (Na_2_HC_6_H_5_O_7_ · 1.5H_2_O), PSA were obtained from Agilent. Ammonium formate (>99 %) was purchased from Fluka (Seelze-Hannover, Germany). Acetic and formic acid (98 % purity) were obtained from Merck (Darmstadt, Germany).

Plant protection products containing active ingredients: azoxystrobin (Amistar 250 SC): boscalid and pyraclostrobin (Signum 33 WG), iprodione (Rovral Aquaflo 500 SC) were used for lettuce spraying.

### Standards

**Fig. 1:**
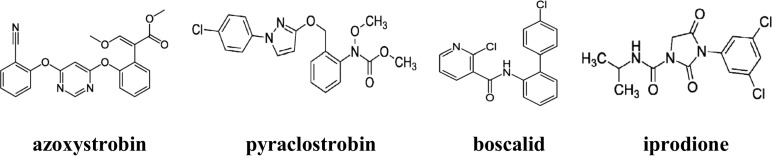
Structure of pesticides (compendium of pesticide common names 2015)

The following pesticides: azoxystrobin, boscalid, iprodione and pyraclostrobin (Fig. 1), were obtained from the Dr. Ehrenstorfer Laboratory (Germany). Pesticide standard stock solutions (purity for all standards >98 % of concentrations) were prepared in acetone and stored at 4 °C. Standard working solutions were prepared by dissolving appropriate amounts of stock solution with a mixture of hexane/acetone (9:1, *v*/*v*).

### Sample preparation of QuEChERS procedure

**Fig. 2:**
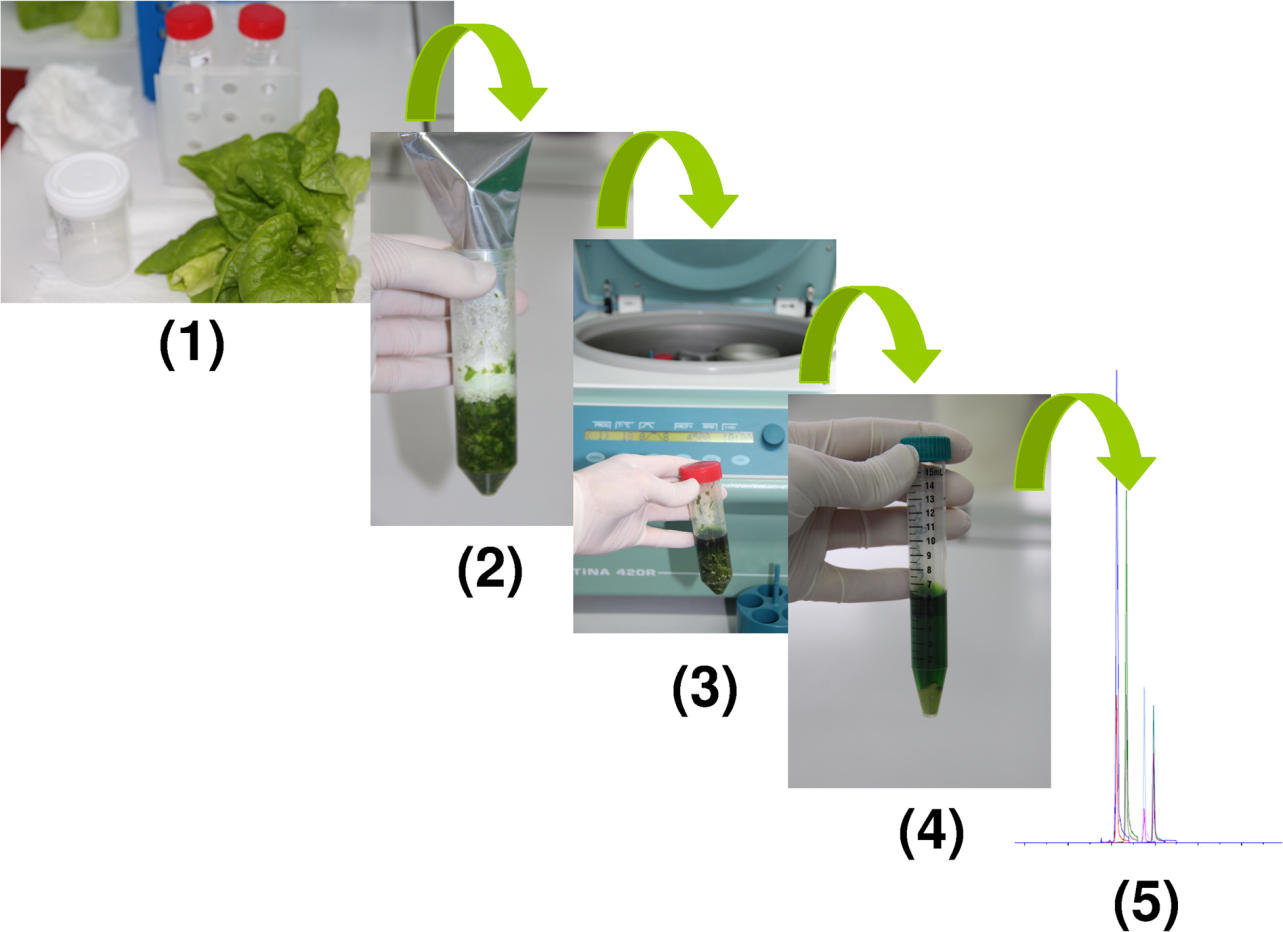
Sample preparation of lettuce and instrumental analysis. Where: 1) Lettuce sample; 2) After vortexing salts containing 4 g MgSO4, 1 g NaCl, 1 g trisodium citrate dihydrate and 0.5 g disodium hydrogen citrate sesquehydrate were added; 3) Vortexed in a vortex-mixer for 5 min; 4) Final extract; 5) LC-MS/MS analysis

To extract four pesticides, 10 g of homogenized lettuce sample was weighed in a 50-mL polypropylene centrifuge tube. The sample was extracted with 10 mL of acetonitrile and vortexed for 5 min using a digital Vortex-Mixer (Velp Scientifica, Usmate, Italy). After vortexing, salts containing 4 g magnesium sulphate, 1 g sodium chloride, 1 g trisodium citrate dihydrate and 0.5 g disodium hydrogen citrate sesquihydrate were added. The tubes were immediately shaken for 1 min, vortexed in a Vortex-Mixer for 5 min at 4500 rpm and then centrifuged at 10,000 rpm (Hettich, Tuttlingen, Germany). The upper layer (acetonitrile extract) was transferred into the dSPE tubes containing 150 mg anhydrous MgSO_4_ and 25 mg PSA. The tubes were vortexed for 30 s and centrifuged at 5000 rpm for 5 min. One millilitre of the final extract was filtered through a 0.2-m hydrophilic PTFE filter, transferred into the appropriately labeled autosampler vial and subsequently analysed using LC–MS/MS (Fig. 2).

### Instrumental analysis LC–MS/MS

An Eksigent Ultra LC-100 (Eksigent Technologies, Dublin, CA, USA) liquid chromatography system was operated at a flow rate of 0.4 ml min^−1^ without split using a SunFire C18 3.5 μm, 2.1 × 100 mm (Waters) analytical column (100 mm × 2.1 mm, 1.8 μm), maintained at 60 °C during the experiments. The volume injected into the LC-MS/MS system was 10 μl. The binary mobile phase consisted of water with 0.5 % acetic acid and 2 mM ammonium formate (phase A) and methanol with 0.51 % formic acid and 2 mM ammonium formate) (phase B).

The initial composition of 99 % A and 1 % B (*v*/*v*) was held for 0.5 min, followed by linear ramping to 75 % of B in 3 min up to 95 % of B in 10 min. After ramping, the mobile phase was returned to the initial composition in 1 min, and this was held for 15 min. The total chromatographic run time was 15.0 min.

### Mass spectrometry conditions

The system MS/MS 6500 QTRAP (AB Sciex Instruments, Foster City, CA) was used for mass spectrometric analysis, equipped with an electrospray ionization source operating in positive ionization mode, set with the following parameters: ion spray voltage (IS), 5000 V; curtain gas, 30 psi; nebulizer gas (GS1), 60 psi; auxiliary gas (GS2), 50 psi; source temperature, 400 °C. Nitrogen was used as the nebulizer and collision gas. Optimization of the compounds was performed by flow injection analysis (FIA), injecting individual standard solutions directly into the source. For quantification, the most intensive MRM transition was selected. Optimized MRM transition parameters: declustering potential (DP), collision energy (CE) and collision cell exit potential (CXP) for each of the compounds, attained in negative ion mode, are presented in Table 1. AB Sciex Analyst software 1.6.2 was used for data acquisition and processing.

**Table 1:**
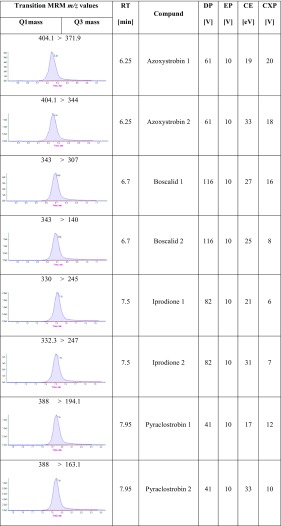
Transition of pesticide (MRM *m/z* values) in lettuce matrix in LC-MS/MS

*Q* quadrupole, *RT* retention time, *EP* entrance potential, *DP* declustering potential, *CE* collision energy, *CXP* collision cell exit potential

### Validation of the method

The validation study was carried out using lettuce samples previously checked to be free of four pesticides: azoxystrobin, boscalid, iprodione and pyraclostrobin. The validation of the method was performed according to Document No. SANCO/12495/2011 (2015). We evaluated the parameters such as: matrix effect, linearity, limits of detection (LOD) and quantification (LOQ), recovery and precision.

The calibration curves were obtained from matrix-matched four-level calibration solutions. Calibration standards were prepared by adding the respective spiking solutions to a blank matrix of lettuce to produce a final concentration 0.001, 0.05, 0.10, 0.50 and 2.0 mg kg^−1^. Linearity was determined from the coefficients of determination (*R*^2^). The recovery data were obtained in three ranges of certain pesticide concentrations in the matrix. The accuracy and precision of the method were evaluated by performing recovery studies. The precision was expressed as the relative standard deviation (RSD). The accuracy can be measured by analysing samples with known concentration and comparing the measured values with the true values. The LOQs were defined as the minimum concentration of the analyte that can be quantified with acceptable accuracy and precision. LODs were calculated using signal-to-noise ratio (S/N) criteria, which was in all cases LOD = 3 S/N.

### Plant material

In this study, leaves of lettuce plants were analysed. The study was conducted from July to November under controlled conditions in phytotrons (Pol-Eko KK 1450 TCP+) in Białystok, podlaskie voivodeship (53,139 N, 23,159 E). The plants were transplanted on a peat culture substrate 21 days after seed germination, and the temperature was maintained at 15–20 °C for further growth of lettuce (3 months). Before harvest, four selected active ingredients (azoxystrobin, boscalid, pyraclostrobin and iprodione) were applied and EM (1%EM and/or 10%EM) and/or yeast *Saccharomyces cerevisiae* 5 % (Y) was applied to each solution. Representative leaves of lettuce samples were collected on day 1, 2, 3, 7 and 14 after the spraying of the pesticide. The samples were stored in individual polyethylene bags in a refrigerator at −20 °C until being analysed.

### Microorganisms

In this study, we used commercial *S. cerevisiae* was bought in the nearest food market for fermentation and the following effective microorganisms: *Lactobacillus plantarum*, L. casei and *Streptoccus lactis* (lactic acid bacteria)*, Rhodopseudomonas palustrus* and *Rhodobacter spaeroides*, (photosynthetic bacteria), *Saccharomyces cerevisiae* and *Candida utilis* (yeasts), *Streptomyces albus* and *Streptomyces griseus* (actinomycetes), and *Aspergillus oryzae, Penicillium sp.* and *Mucor hiemalis* (fermenting fungi).

### Degradation in plants under field conditions (dynamics)

The decline of pesticide concentration in time is often described according to first-order kinetics (Beulke and Brown 2001) and can be expressed as C_t_ = C_0_∙e^-kt^, where: C_t_ represents the concentration at the time of t [mg kg^−1^]; C_0_ represents the concentration at the time zero t = 0 [mg kg^−1^], initial deposits; *t* is time; and *k* is the degradation rate constant in days^−1^. The half-life (t_(1⁄2)_) was calculated from the k values for each experiment t_(1⁄2)_ = ln2⁄k. The theoretical degradation time (t_0.01_) up to the level of 0.01 mg kg^−1^ or below was determined according t_0,01_ = ln(0.01⁄C_0_)/(−k). All the experiments were carried out at least in duplicate and standard deviations were calculated.

### Chronic (long-term) exposure assessment

The health risk estimation was calculated through the comparison of found residues with the established acceptable daily intake (ADI). The level of residue concentration in a product was determined as the arithmetic mean of all the results obtained. The results under LOD of analytical methods used for intake calculations were taken as LOD values. The values of ADI are elaborated by the Joint FAO/WHO Meeting on Pesticides Residues, European Food Safety Authority (EFSA) of European Union or Federal Institute for Risk Assessment (BfR 2006). Germany. The long-term (chronic) dietary consumer exposure to pesticide residues was estimated by using an EFSA calculation model Pesticide Residue Intake Model “PRIMo” revision 2 (Heusinkveld et al. 2013). based on national food consumption, unit weights and internationally agreed risk assessment methodologies to assess the long-term (chronic) exposure of consumers, accepting consumption at the level of the 97.5 percentile (GEMS/FOOD 2012). For lettuce, the methodology described below has been used, where the piece of vegetable may contain a higher residue than composite samples from residue trials (unit weight > 25 g). The international estimated short-term intake was calculated according to equation: IESTI = LP × (HR–P) × v/bw, where: U is the unit weight of individual items of the commodity in kilograms, HR–P is the highest residue level in milligram per kilogram, v is the variability factor applied to the composite residue to approximate the residue level in a high-residue single unit (depending on the commodity, lettuce v = 5), LP is the 97.5th percentile of portion sizes taken by people consuming the commodity in kilogram of food per day, bw is the mean body weight for the target population subgroup in kilogram.

The international estimated daily intake (IEDI) of pesticide residues was calculated as follows: IEDI = ∑(Fi × RLi)/mean bw where: IEDI is the international estimated daily intake, Fi is the food consumption data, RLi is the residue level to the commodity.

The long-term risk assessment of the intakes compared to the pesticide toxicological data was performed for (groups: cluster D, adults and children) by calculating the hazard quotient (HQ), by dividing the international estimated daily intake with the relevant acceptable daily intake which are considered to be safe levels of exposure over the lifetime: HQ = IEDI/ADI × 100 % where: ADI is the acceptable daily intake.

## Result and discussion

### Validation of the method

Mean recoveries and relative standard deviations (RSD) ranged from 75 to 112 % with relative standard deviation below 15 %. The linearity of the method was evaluated with matrix-matched calibration curves and was good showing *R*^2^ ≥ 0.9998. The LOQ was 0.001 mg kg^−1^ and LOD being 0.0003 mg kg^−1^, respectively. Matrix effect (ME) for all the analytes was observed, and ME values ranged from −37.5 ± 8.1 to 45.9 ± 7.9 %. The positive and negative values of the ME% reflect matrix-induced enhancement and suppression, respectively. Matrix effect was compensated by using the matrix-matched lettuce samples. These results showed that the validation parameters were good; thus, target pesticides were satisfactorily determined in lettuce samples.

### Pesticide degradation

The obtained data in Table 2 show the degradation of azoxystrobin, boscalid, pyraclostrobin and iprodione in the lettuce cultivated. It was revealed that after the application of azoxystrobin, boscalid, pyraclostrobin and iprodione, the initial amounts were 0.64, 1.23, 2.30 and 0.35 mg kg^−1^ in the lettuce cultivated, respectively. These figures decreased gradually till they reached 0.08, 0.08, 0.19 and 0.04 mg kg^−1^ after the 14 days of application. Among the tested substances, the biggest degradation percentage was observed in boscalid, approximately 94 %, whereas the smallest in azoxystrobin about 87 % after the 14 days of application. Humbert et al. (2007) and Juraske et al. (2007) point out that to address the challenge of measuring the dynamic behaviour of pesticides in/on plants, it is important to take into account the time between pesticide application and harvest in order to better estimate the pesticide half-life in/on plants.

**Table 2:** The degradation (in %) of azoxystrobin, boscalid, iprodione and pyraclostrobin in the lettuce cultivated under controlled conditions

Periods	Active substances							
	Azoxystrobin		Pyraclostrobin		Boscalid		Iprodione	
	mg kg^−1^	Loss %	mg kg^−1^	Loss %	mg kg^−1^	Loss %	mg kg^−1^	Loss %
1 day	0.64	–	0.35	–	1.23	–	2.30	–
2 days	0.42	34.0	0.28	19.9	0.95	22.4	1.53	33.5
3 days	0.22	65.0	0.16	55.3	0.62	49.4	0.88	61.7
7 days	0.11	82.8	0.07	81.0	0.23	81.2	0.51	77.8
14 days	0.08	87.4	0.04	89.2	0.08	93.9	0.19	91.9

**Fig. 3:**
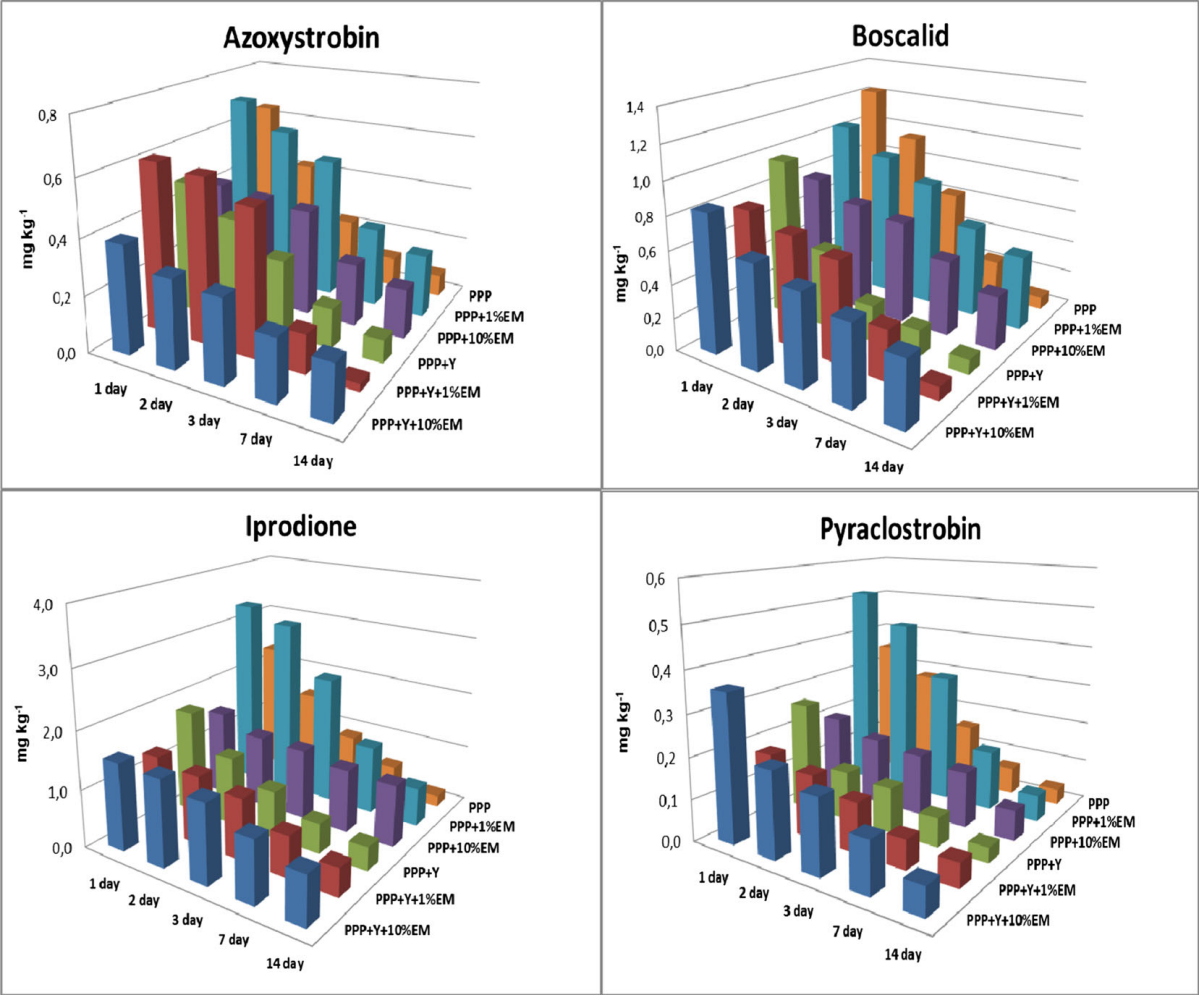
The dynamics of degradation of fungicide: azoxystrobin, boscalid, pyraclostrobin and iprodione after 1, 2, 3, 7 and 14 day in the lettuce leaves with the addition of effective microorganisms (1 % and 10%EM) and yeast (Y)

Figure 3 shows the dynamics of degradation of azoxystrobin, boscalid, pyraclostrobin and iprodione in the lettuce leaves as pure substance and with the addition of EM and yeast. Konstantinos and Suha (2011) report that microorganisms have a big influence on the disappearance of pesticides. Such properties are exhibited to the greatest extent in *Arthobacter* and *Bacillus* bacteria, strains of actinomycetes of *Nocardia* and *Streptomyces* and the fungi belonging to the following genera: *Penicillium, Aspergillus*, *Fusarium* and *Trichoderma*. On the other hand, plants are complex organisms with a variety of biochemical systems composed of a vast number of metabolites with diverse physico-chemical properties. Therefore, sometimes, it is not possible to predict changes in the distribution of PPPs (Aislabie and Lloyd-Jones 1995; Konstantinos and Suha 2011). As can be observed, the addition of yeast (Y) to the tested substances accelerated the decomposition of compounds, and their concentrations were comparable with the individual substances: in the case of pyraclostrobin and boscalid, at the end of the experiment, the percentage of decomposition was at 86.2 and 91.1 %, respectively (Fig. 3).

In the study, the mixtures of Y + 1%EM and Y + 10%EM both stimulated and inhibited the degradation of the tested active substances (Fig. 3). Azoxystrobin residue was observed in the leaves of the lettuce treated with Y + 1%EM at 0.028 mg kg^−1^ (95.4 % degradation), while in the case of iprodione and pyraclostrobin, decomposition was at a level of about 62.56 and 67.58 %, respectively. On the other hand, in the plants treated with a mixture of Y + 10%EM, the dissipation dynamics was at a level of 49.3 % for azoxystrobin, 81.3 % for pyraclostrobin, 53.1 % for boscalid and 45.4 % for iprodione after 14 days of application (Fig. 3). Aislabie and Lloyd-Jones (1995) report that microbial degradation is the primary route for loss and is therefore the key process affecting the dynamics of pesticide residues in the environment, including their persistence, which is not supported by our own research.

In our study, it was observed that the addition of 1%EM and 10%EM to active substances increased the time of dissipation of fungicides in the lettuce. The initial concentration of individual fungicides with 1%EM in the plants was 0.46 mg kg^−1^ for azoxystrobin, 0.52 mg kg^−1^ for pyraclostrobin, 1.04 mg kg^−1^ for boscalid and 3.32 mg kg^−1^ for iprodione. After 14 days, it was reduced to a level of 67.6, 87.6, 57.1 and 79.9 % respectively, compared with the initial deposit. In turn, adding 10%EM to the test substances (Fig. 3) strongly inhibited the degradation of iprodione, and its concentration decreased by 30 %, and in the case of other test substances, degradation was approximately 60 %. Detoxification processes occurring in plants can take place according to different mechanisms. The active agents may be conjugated to natural plant ingredients (e.g. saccharides) to form glycosides. These compounds may also be subject to enzymatic hydroxylation and conjugation with glutathione. Such examples are sorghum and maize, which have transferase isoenzymes capable of detoxifying atrazine (Aislabie and Lloyd-Jones 1995; Van Erd et al. 2003). There is also a possibility of binding pesticides and their metabolites in permanent connections with the components of cell walls to form a so-called residue bound (Van Erd et al. 2003).

### Relationship between physico-chemical parameters and half-life

The chemical structure of pesticides has specific transfer and permeability properties to cross through plant cuticles. The permeation through plant cuticles depends on the solute mobility in the limiting skin, the path length of the limiting skin, the partition coefficient between cuticle and deposited surface residue. The octanol-water partition coefficient log *P* is a key parameter in the studies of the environmental fate of chemical substances. It is a useful parameter in the prediction of adsorption behaviour of pesticides (Trapp 2004, Schreiber 2005).

**Fig. 4:**
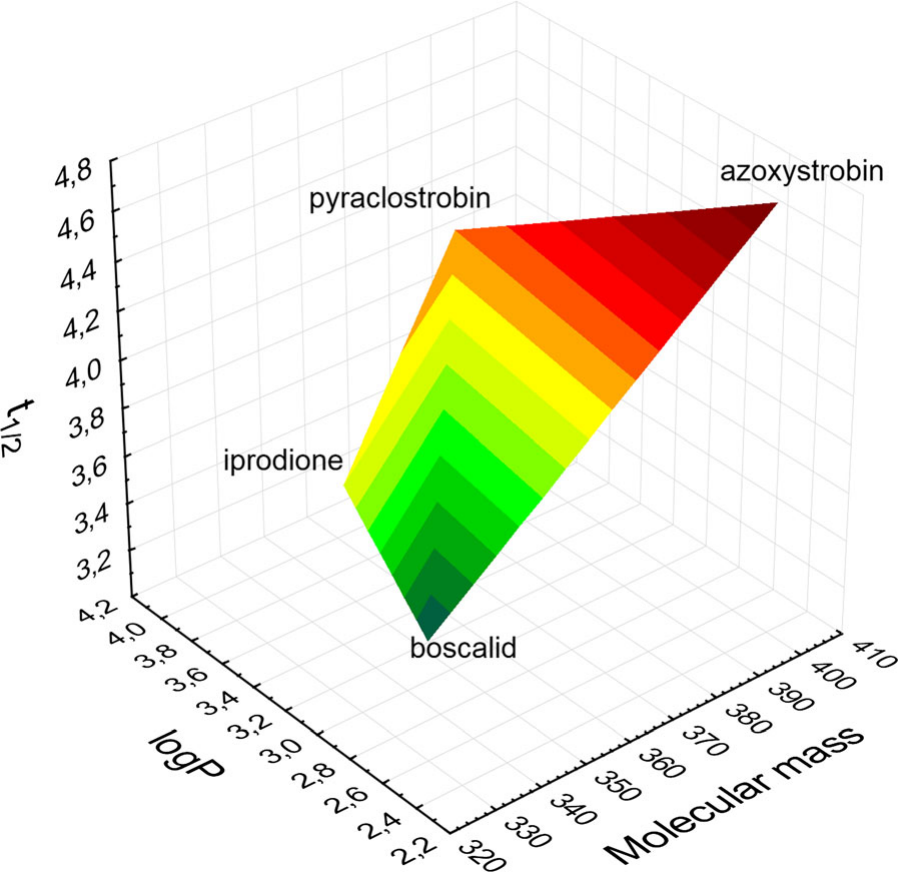
The relationship between of four fungicides and the molecular mass, the log *P* coefficient and experimental half-life

Figure 4 shows the relationship between of four fungicides and the molecular mass, the log *P* coefficient and experimental half-life. Each of the substances tested has a different molecular weight. The compounds of high molecular weight, such as azoxystrobin, decompose slowly and the ones of low molecular weight, such as boscalid, decompose faster. Iprodione and pyraclostrobin have the same coefficient log P, but their molecular weights decide about the decomposition rate, and pyraclostrobin decomposes slower than iprodione. According to Zhang et al. (2010). in the process of foliar application of PPPs, a construction area of the plant plays an important role in retaining the residue. The plants covered with hairs accumulate significant amounts of liquid and powdery preparations. The wax layer covering the surface of some plants may impede the penetration of PPPs, especially those soluble in water. In the case of the compounds of hydrophobic character, it is expected that they will easily penetrate and accumulate in the wax layer, like in the fatty tissue of animals (Kah et al. 2007; Swarcewicz and Gregorczyk 2012; Bagi et al. 2014).

### The degradation kinetics

The average residue concentrations of azoxystrobin on leafy vegetables ranged from 0.642 mg kg^−1^ on the first day to 0.081 mg kg^−1^ after 14 days, which accounted for approximately 12.0 % of the initial residues. The degradation kinetics of azoxystrobin deposits was well described by a first-order decay equation *R*^2^ = 0.81 (Table 3). For boscalid, it ranged from 1.23 mg kg^−1^ on the first day to 0.08 mg kg^−1^ after 14 days (6 % of the initial residues) and a first-order kinetics was *R*^2^ = 0.98. The mean values of iprodione residue after 24 h of application were at the level of 2.30 mg kg^−1^. Then, dissipation occurred according to the equation: *R*^2^ = 0.94 and on 14 days, they were reduced to about 8.1 % of the initial value and amounted to 0.19 mg kg^−1^. The average value of pyraclostrobin on leafy vegetables ranged from 0.35 mg kg^−1^ on the first day and then decreased in accordance with the exponential equation: *R*^2^ = 0.9, then 0.04 mg kg^−1^ after 14 days (Table 3), which amounted to about 10.8 % of the initial residues. Among the active substances analysed, the highest residue to the initial value was detected in the lettuce leaves treated with azoxystrobin.

**Table 3:** The statistical parameters derived from the exponential trendline of the data degradation for azoxystrobin, boscalid, iprodione and pyraclostrobin residues in lettuce

	Active substances							
	Azoxystrobin		Pyraclostrobin		Boskalid		Iprodione	
	*R* ^2^	C(t)	*R* ^2^	C(t)	*R* ^2^	C(t)	*R* ^2^	C(t)
PPP+ Y + 10%EM	0.86	0.36e^−0.048x^	0.90	0.29e^−0.112x^	0.81	0.73e^−0.048x^	0.98	1.59e^−0.048x^
PPP+ Y + 1%EM	0.99	0.91e^−0.25x^	0.88	0.17e^−0.086x^	0.99	0.96e^−0.171x^	0.97	1.36e^−0.076x^
PPP + Y	0.93	0.46e^−0.132x^	0.85	0.19e^−0.127x^	0.78	0.63e^−0.156x^	0.81	1.42e^−0.103x^
PPP + 10%EM	0.93	0.45e^−0.071x^	0.96	0.20e^−0.071x^	0.97	0.78e^−0.064x^	0.66	1.37e^−0.021x^
PPP + 1%EM	0.90	0.69e^−0.087x^	0.98	0.56e^−0.161x^	0.91	0.99e^−0.062x^	0.96	3.53e^−0.126x^
PPP	0.81	0.49e^−0.148x^	0.90	0.33e^−0.169x^	0.98	1.32e^−0.214x^	0.94	2.07e^−0.179x^

*R* ^2^ correlation coefficients, *C(t)* represents the concentration at the time

### Value of half-life and T_0.01_ for four fungicides

Metabolism and degradation half-lives or rate constants of pesticides in/on plants are the key data required for the assessment of PPPs (Humbert et al. 2007). According to our experiment results, the half-life of azoxystrobin was 4.7 days; for boscalid, it was 3.2 days, for iprodione 3.9 days and for pyraclostrobin 4.1 days if applied on leafy vegetables (Table 4). In addition, half-life of azoxystrobin in the lettuce leaves treated additionally with EM 1 % and 10 %, and yeast 5 % was from 2.7 to 14.4, for boscalid was from 4.4 to 14.4, for iprodione was from 5.5 to 33.0 and for pyraclostrobin was from 4.3 to 9.8 (Table 4). According to Navarro et al. (2007). the decomposition of pesticide occurs mainly as a result of photochemical, chemical and biochemical reactions, such as oxidation, reduction, hydrolysis, interaction with free radicals or nucleophilic substitution by means of water. They are often catalysed by soil constituents (e.g. cations of some metals (Fe, Cu), aluminum oxide) or organic compounds (Zhang et al. 2010). The final value of PPP residue in plants is affected by climatic conditions such as intensive ultraviolet radiation, air temperature, precipitation and wind.

**Table 4:** The half-life (t_1/2_) and the time after which the residue has reached a level of 0.01 mg kg^−1^ (t _0.01_) for azoxystrobin, boscalid, iprodione and pyraclostrobin in lettuce

Treatments	Active substances							
	Azoxystrobin		Pyraclostrobin		Boscalid		Iprodione	
	t_1/2_	t_0.01_	t_1/2_	t_0.01_	t_1/2_	t_0.01_	t_1/2_	t_0.01_
	day	day	day	day	day	day	day	day
PPP+ Y + 10%EM	14.4	74.0	6.2	30.0	14.4	89.0	14.4	105.5
PPP+ Y + 1%EM	2.7	18.0	8.1	33.0	4.1	27.0	9.1	65.0
PPP + Y	5.3	29.0	5.5	23.0	4.4	26.5	6.7	48.0
PPP + 10%EM	9.8	53.5	9.8	42.0	10.8	68.0	33.0	234.0
PPP + 1%EM	8.0	52.0	4.3	25.0	11.2	74.0	5.5	46.5
PPP	4.7	26.0	4.1	21.0	3.2	23.0	3.9	29.5

A calculation of the theoretical time (t _0.01_), in which it is possible to obtain the concentration of 0.01 mg kg^−1^, acceptable in food for children was performed. On the basis of the results, it appears that at the time of harvest, the residue in the lettuce leaves was higher than the required limit for raw materials for the production for young children (Tab. 4). The analysed pure active substances and those with the addition of effective microorganisms and yeasts reach a level of 0.01 mg kg^−1^ for azoxystrobin between day 18 and 74, for boscalid between day 23 and 89, for iprodione between day 29.5 and 234.0 and for pyraclostrobin between day 21 and 42.0 (Table 4). According to Ndona et al. (2011). plants, unlike animals, have little opportunity to excrete waste products. The only way is the transpiration system of the leaves. The fate of PPPs residue in the plants is not affected to a greater extent, by the mechanism relying on the expulsion of PPPs residue from the plants. A more important role is played by conversions catalysed by a number of plant enzymes, such as hydrolysis reactions of oxidation and subsequent coupling. The transformation products, e.g. fragments of the parent molecule, are among others used by the plant for the synthesis of amino acids (Zhang et al. 2010).

### Risk assessment

Table 5 shows the results of the evaluation of the health risks of exposure of children and the elderly related to the consumption of lettuce containing the residues of PPPs. The food consumption rate for the lettuce in British children is 0.021, in adults 0.117 g/kg/person/day, and for WHO cluster diet E (Austria, Belgium, Croatia, Czech Republic, Denmark, France, Germany, Hungary, Ireland, Luxemburg, Malta, Netherlands, Poland, Slovakia, Slovenia, Switzerland, United Kingdom of Great Britain and Northern Ireland), it is at 0.198 g/kg/person/day. The consumer risk is routinely evaluated as a part of the approval process for pesticides and is based on residue trials. The approval of a pesticide is only recommended when the consumer risk is acceptable (Heusinkveld et al. 2013; U.S. Environmental Protection Agency 2002). The risk assessments for a single detected pesticide in the lettuce collected on the first, second, third, seventh and fourteenth day after the application were performed by estimating the hazard quotient (HQ), calculated by dividing the exposure by the acceptable daily intake (ADI) for individual pesticides. Since the HQ method assumes the same type of adverse effect for all of the detected pesticides, it is a relatively conservative approach to cumulative risk assessment. The HQ_S_ for the individual pesticides ranged from 0.4 to 64.8 % on day1, but after 14 days, it ranged from 0.0 to 20.9 % for children and adults, respectively. It indicated no risk of adverse effects following exposure to individual pesticides and their mixtures with EM and yeast. As shown in Table 5, the consumer exposure to pesticides does not exceed the value of 100 % of the ADI. Boscalid and pyraclostrobin are contact pesticides, thus they stay on the surface of leaves. In addition, strobilurins and carboxamide exhibit irritating effects on eyes, skin or the respiratory tract, but there is no data on their mutagenic potential, or the impact that may have on the endocrine system (Pesticide Properties DataBase 2014). They have the lowest ADI = 0.04 and 0.03 mg kg^−1^ respectively. Nevertheless, it is recommended to continue monitoring azoxystrobin, boscalid, pyraclostrobin and iprodione residues in food commodities.

**Table 5:** Estimation of chronic dietary exposure to pesticide residue based on average residues detected in lettuce cultivated obtained after 1, 2, 3, 7 and 14 days

	ADI [mgkg^−1^ b.w./d]	R [mg kg^−1^] Day 1	HQ (%)			R [mg kg^−1^] Day 2	HQ (%)			R [mg kg^−1^] Day 3	HQ (%)			R [mg kg^−1^] Day 7	HQ (%)			R [mg kg^−1^] Day 14	HQ (%)		
			UK	UK	WHO cluster diet E		UK	UK	WHO cluster diet E		UK	UK	WHO cluster diet E		UK	UK	WHO cluster diet E		UK	UK	WHO cluster diet E
			Toddler	Adult			Toddler	Adult			Toddler	Adult			Toddler	Adult			Toddler	Adult	
Boscalid (MRL 30 mg kg^−1^)																					
PPP + Y + 10%EM	0.04	0.84	4.3	24.5	41.5	0.63	3.2	18.3	31.0	0.55	2.9	16.2	27.5	0.48	2.5	14.1	23.9	0.39	2.0	11.5	19.5
PPP + Y + 1%EM		0.76	3.9	22.2	37.5	0.68	3.5	19.9	33.7	0.61	3.1	17.8	30.2	0.31	1.6	9.0	15.2	0.08	0.4	2.5	4.2
PPP + Y		0.97	5.0	28.3	47.8	0.47	2.4	13.8	23.4	0.22	1.1	6.4	10.9	0.17	0.9	4.9	8.2	0.09	0.4	2.5	4.3
PPP + 10%EM		0.78	4.0	22.7	38.5	0.67	3.4	19.6	33.2	0.62	3.2	18.2	30.9	0.45	2.3	13.3	22.5	0.33	1.7	9.5	16.1
PPP + 1%EM		1.04	5.4	30.6	51.8	0.89	4.6	26.2	44.3	0.77	4	22.7	38.4	0.56	2.9	16.3	27.7	0.45	2.3	13.1	22.2
PPP		1.22	6.3	35.9	60.7	0.95	4.9	27.8	47.1	0.62	3.2	18.1	30.7	0.23	1.2	6.8	11.4	0.08	0.4	2.2	3.7
Azoxystrobin (MRL 15 mg kg^−1^)																					
PPP + Y + 10%EM	0.2	0.39	0.4	2.3	3.8	0.31	0.3	1.8	3.1	0.3	0.3	1.7	3.0	0.22	0.2	1.3	2.2	0.2	0.2	1.1	1.9
PPP + Y + 1%EM		0.61	0.6	3.6	6.0	0.59	0.6	3.4	5.8	0.52	0.5	3.0	5.2	0.14	0.1	0.8	1.4	0.03	0.0	0.2	0.3
PPP + Y		0.48	0.5	2.8	4.8	0.38	0.4	2.2	3.8	0.27	0.3	1.6	2.7	0.14	0.1	0.8	1.4	0.08	0.1	0.5	0.8
PPP + 10%EM		0.42	0.4	2.5	4.2	0.4	0.4	2.3	3.9	0.39	0.4	2.3	3.8	0.23	0.2	1.3	2.2	0.18	0.2	1.0	1.8
PPP + 1%EM		0.7	0.7	4.1	7.0	0.6	0.6	3.5	6.0	0.52	0.5	3.0	5.1	0.29	0.3	1.7	2.9	0.23	0.2	1.3	2.3
PPP		0.64	0.7	3.8	6.4	0.42	0.4	2.5	4.2	0.22	0.2	1.3	2.2	0.11	0.1	0.6	1.1	0.08	0.1	0.5	0.8
Iprodione (MRL 10 mg kg^−1^)																					
PPP + Y + 10%EM	0.06	1.52	5.2	29.7	50.4	1.49	5.1	29.1	49.4	1.37	4.7	26.7	45.2	1.06	3.6	20.8	35.2	0.83	2.9	16.2	27.5
PPP + Y + 1%EM		1.31	4.5	25.6	43.4	1.2	4.1	23.4	39.6	1.07	3.7	20.9	35.3	0.71	2.4	13.9	23.5	0.49	1.7	9.6	16.2
PPP + Y		1.8	6.2	35.2	59.5	1.18	4.1	23.1	39.1	0.82	2.8	16.0	27.1	0.52	1.8	10.2	17.3	0.4	1.4	7.8	13.2
PPP + 10%EM		1.51	5.2	29.5	49.9	1.26	4.3	24.5	41.5	1.23	4.2	24.1	40.8	1.1	3.8	21.4	36.3	1.07	3.7	20.9	35.4
PPP + 1%EM		3.32	11.4	64.8	109.7	3.09	10.6	60.4	102.3	2.24	7.7	43.7	74.1	1.19	4.1	23.3	39.5	0.67	2.3	13.0	22.0
PPP		2.3	7.9	44.9	76.0	1.53	5.2	29.8	50.5	0.88	3.0	17.2	29.1	0.51	1.8	10.0	16.9	0.19	0.6	3.6	6.2
Pyraclostrobin (MRL 2 mg kg^−1^)																					
PPP + Y + 10%EM	0.03	0.36	23.5	1.6	13.9	0.21	1.4	8.0	13.6	0.18	1.2	6.9	11.8	0.12	0.8	4.8	8.1	0.07	0.5	2.6	4.4
PPP + Y + 1%EM		0.18	11.6	0.8	6.9	0.15	1.0	6.0	10.1	0.12	0.8	4.7	7.9	0.07	0.5	2.7	4.6	0.06	0.4	2.2	3.8
PPP + Y		0.26	17.5	1.2	10.3	0.12	0.8	4.7	7.9	0.11	0.7	4.3	7.2	0.07	0.5	2.7	4.6	0.04	0.2	1.4	2.4
PPP + 10%EM		0.2	13.1	0.9	7.7	0.17	1.1	6.4	10.9	0.15	1.0	5.9	9.9	0.13	0.9	5.3	8.9	0.07	0.5	2.8	4.8
PPP + 1%EM		0.52	34.2	2.3	20.2	0.44	3.0	17.2	29.2	0.32	2.2	12.4	21.1	0.15	1.0	5.7	9.7	0.06	0.4	2.5	4.3
PPP		0.35	23.1	1.5	13.6	0.28	1.9	10.9	18.5	0.16	1.1	6.1	10.3	0.07	0.5	2.6	4.4	0.04	0.3	1.5	2.5

MRL (from EU Pesticides database from lettuce), *ADI* acceptable daily intake, *HQ* hazard quotient, *R* residue

## Conclusions

1. For all tested fungicides and their mixtures with addition of effective microorganism and yeast, the residues of fungicides in the lettuce leaves were at least five times lower than the established MRL.
2. The addition of effective microorganisms to analysed fungicides resulted in the significant inhibition of decomposition, strengthening the final result and maintaining the active substance at a very high level, particularly in iprodione.
3. The addition of yeast stimulated the distribution of pyraclostrobin and boscalid in the lettuce leaves.
4. The health risk analysis revealed that azoxystrobin, boscalid, pyraclostrobin and iprodione without and with the addition of effective microorganism and yeast do not pose a direct hazard to human health in particular in children, in spite of its presence in the cultivated lettuce.
5. Among the active substances analysed after 14 days, none reached the limit fixed for raw materials for the production for small children at level of 0.01 mg kg^−1^.
